# Supplementary material for: Cancer expression quantitative trait loci (eQTLs) can be determined from heterogeneous tumor gene expression data by modeling variation in tumor purity
Source: Genome Biol. 2018 Sep 11;19:130. doi: 10.1186/s13059-018-1507-0 (PMC6131897; doi:10.1186/s13059-018-1507-0)
Supplement: Supplementary file 2 — Model derivation: a derivation of the “interaction model” used to find cancer eQTLs. (PDF 110 kb) [file 13059_2018_1507_MOESM2_ESM.pdf]

Derivation of an "interaction model" for identifying cell-type specific eQTLs from gene expression data generated on 2 mixtures of cell types, when cell type proportions are known

July 27, 2018

This derivation uses terminology referring to "cancer" and "normal" cell types, but is applicable to any mixture of 2 cell types.

We assume that bulk gene expression (in this case from tumors) is a weighted sum of the gene expression in underlying cell types (in this case cancer and normal cells). This assumption has been demonstrated to be reasonable by Shen-Orr *et al.* (Nature Methods, 2010):

$$y = (1-p)c + pn \tag{1}$$

where y is a gene expression value in an individual in the bulk tissue (e.g. tumor), c is expression in one cell type (e.g. cancer cells), n is expression in the second cell type (e.g. tumor-associated normal cells) and p is the proportion of total mRNA contributed by the second cell type—in this case approximated by the proportion of tumor-associated normal cells (i.e. $p = 1$ if the sample has 100% normal cells, $p = 0$ if 100% cancer cells). We estimated these proportions using the CPE method (Aran *et al.*, Nature Communications, 2016).

We assume:

$$c = \beta_{0_c} + \beta_c x + \epsilon_c \tag{2}$$

$$n = \beta_{0_n} + \beta_n x + \epsilon_n \tag{3}$$

where β_c and β_n are eQTL effects influencing gene expression as a function of genotype x in cancer cells and normal cells respectively. β_{0_c} and β_{0_n} are constant terms and ϵ_c and ϵ_n are the other sources of variation.

Substituting c and n in (1) for the values from (2) & (3) gives:

$$y = (1-p)(\beta_{0_c} + \beta_c x + \epsilon_c) + p(\beta_{0_n} + \beta_n x + \epsilon_n)$$

$$y = \beta_{0_c} - p\beta_{0_c} + p\beta_{0_n} + \beta_c x - p\beta_c x + p\beta_n x + \epsilon_c - p\epsilon_c + p\epsilon_n$$

$$y = \beta_{0_c} + \beta_c x + (\beta_{0_n} - \beta_{0_c})p + (\beta_n - \beta_c)(p \times x) + \epsilon_c - p\epsilon_c + p\epsilon_n$$

Additionally, correcting for expression heterogeneity and population structure, we include the following covariates:

$$
\begin{aligned}
y = \beta_{0_c} + \beta_{0_{ab}} + \beta_c x + \boldsymbol{\beta_2} \cdot \boldsymbol{a} + \boldsymbol{\beta_3} \cdot \boldsymbol{b} + \\
(\beta_{0_n} - \beta_{0_c})p + (\beta_n - \beta_c)(p \times x) + \epsilon_{ab} + \epsilon_c - p\epsilon_c + p\epsilon_n
\end{aligned}
$$

where $\boldsymbol{a}$ is the first 3 principal components of the genotype matrix to estimate population structure, $\boldsymbol{b}$ is 35 PEER factors, $\beta_{0_{ab}}$ and ϵ_{ab} are the constant and residual variation introduced by including these terms. Bold typeface denotes a vector. Note: there is an assumption here that population structure affects gene expression in the same way in both cell types; thus, we generally recommend applying this approach to reasonably homogeneous groups of individuals.

Group the constant (intercept) and residual terms, such that:

$$
\begin{aligned}
\beta_0 &= \beta_{0_c} + \beta_{0_{ab}} \\
\epsilon &= \epsilon_{ab} + \epsilon_c - p\epsilon_c + p\epsilon_n
\end{aligned}
$$

Thus:

$$
y = \beta_0 + \beta_c x + \boldsymbol{\beta_2} \cdot \boldsymbol{a} + \boldsymbol{\beta_3} \cdot \boldsymbol{b} + (\beta_{0_n} - \beta_{0_c})p + (\beta_n - \beta_c)(p \times x) + \epsilon
$$

and let:

$$
\begin{aligned}
\beta_4 &= \beta_{0_n} - \beta_{0_c} \\
\beta_5 &= \beta_n - \beta_c \\
\beta_1 &= \beta_c
\end{aligned}
$$

which yields [EQ2] from the main text:

$$
y = \beta_0 + \beta_1 x + \boldsymbol{\beta_2} \cdot \boldsymbol{a} + \boldsymbol{\beta_3} \cdot \boldsymbol{b} + \beta_4 p + \beta_5 (p \times x) + \epsilon
$$

Assuming:

$$
\epsilon \sim N(0, \sigma^2)
$$

these parameters—including the cancer cell-specific eQTL effect β_1—can be estimated by e.g. ordinary least squares or maximum likelihood estimation.

Notably, the correct interpretation of the interaction term ($\beta_5 = \beta_n - \beta_c$) is that it represents an estimate of the difference in the eQTL effect between the two underlying cells types (here referred to as cancer and normal).
