# Supplementary material for: Cancer expression quantitative trait loci (eQTLs) can be determined from heterogeneous tumor gene expression data by modeling variation in tumor purity
Source: Genome Biol. 2018 Sep 11;19:130. doi: 10.1186/s13059-018-1507-0 (PMC6131897; doi:10.1186/s13059-018-1507-0)
Supplement: Supplementary file 3 — Supplementary figures. (PDF 2475 kb) [file 13059_2018_1507_MOESM3_ESM.pdf]

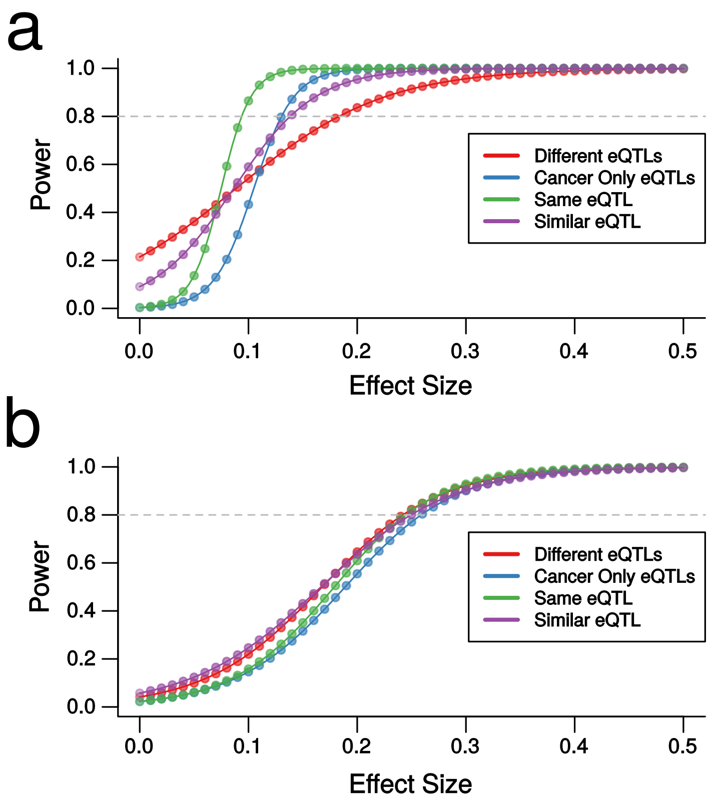

Figure S1: Power estimates for (a) the conventional and (b) the interaction model. The x-axis represents effect size and the y-axis the estimated power to recover such an effect using either model. The curves to estimate power for a given effect size were generated by fitting the following logistic regression model: $p(x) = \frac{1}{1+e^{-(\beta_0+\beta_1 x)}}$ where $p(x)$ is the estimate of the power identify a true positive association at an effect size x. These estimates were generated from the simulated data presented in Results; the points represent the fitted values of the training data. The horizontal dashed gray line represents 80% power to recover a given effect. In simulation the conventional model is slightly more likely to recover true positive associations, but at the cost of potentially vast numbers of false positive associations arising from normal cells (as outlined in Results).

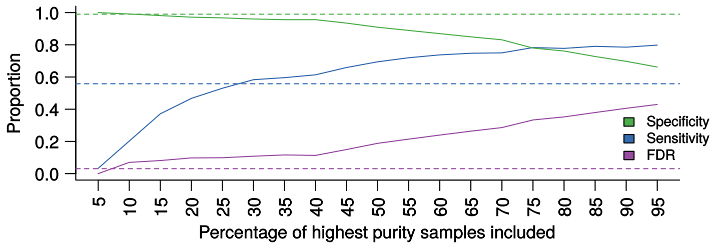

Figure S2: Solid lines show estimates of the sensitivity, specificity and FDR when the proportion of samples used to map eQTLs using a conventional approach is increased from 5% to 95% in the simulated data. The x-axis represents the proportion of samples that were included in the analysis. Samples are included based on their estimated proportion of cancer cells. For example, at a value of "30" on the x-axis, the 30% of samples with the highest cancer cell content have been used in the eQTL mapping. Notably, in these simulations, the true FDR is exceeded almost immediately following inclusion of samples with anything but negligible normal cell content and sensitivity lags far behind the performance achieved by interaction model (shown as the dashed lines).

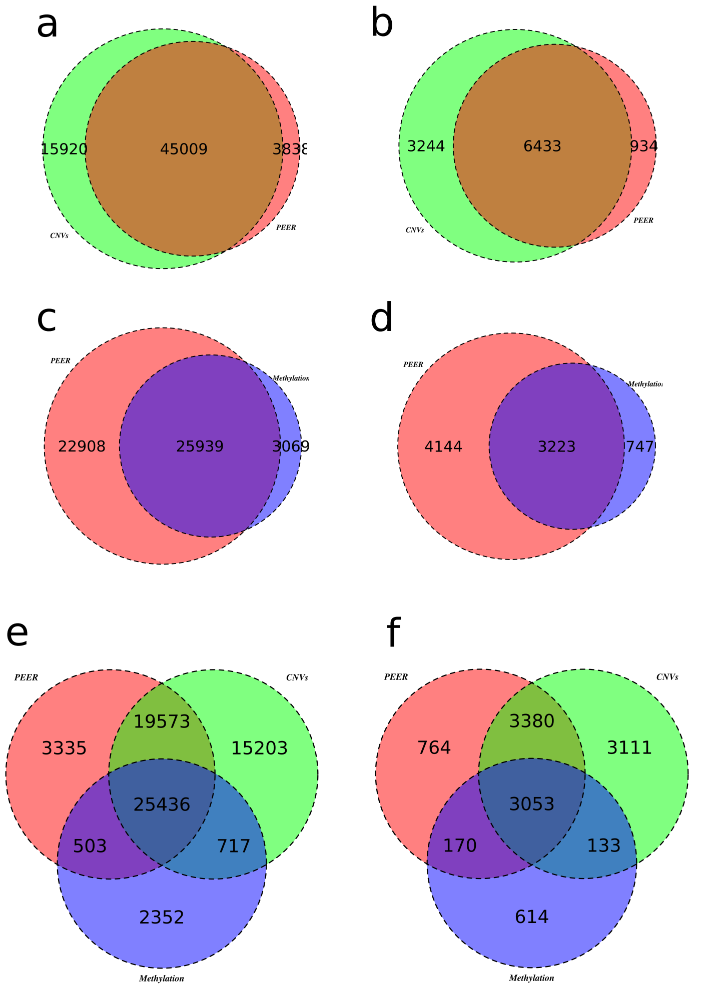

Figure S3: Li et al. (PMID:23374354) previously performed an eQTL analysis in TCGA breast cancer samples, controlling for somatic copy number and promoter methylation status. These Venn diagrams show the overlap of significant eQTLs ($FDR < 0.05$) identified using a conventional approach (left, Fig. a, c, e) and the interaction model (right, Figs b, d, f). Including an additional covariate (over Eqs. 1 & 2 in the main text) for somatic copy number (Fig. a & b) slightly increases the number of eQTLs identified using either approach, whereas methylation status (Fig. c & d) causes a substantial decrease in the number of eQTLs identified. Thus, in general, controlling for somatic copy number likely improves power over estimating hidden confounding factors from expression heterogeneity (i.e. PEER factors) alone. However, controlling for methylation negatively affects model performance, which we would have expected given the likely shared mechanisms of many methylation and expression QTLs (PMID:29476079). In conclusion, we do not recommend including methylation as a model covariate in cancer eQTL mapping and suggest that it may be beneficial to control for somatic copy number, but note that this will require a matrix inversion for each SNP-gene pair; hence, this comes at a substantial (at least 10 fold) computational cost. Additionally, in controlling for somatic copy number, one would lose power to detect cancer eQTL associations that arise if there is a tendency for some copy number variants to develop on a specific germline background (PMID:28188128).

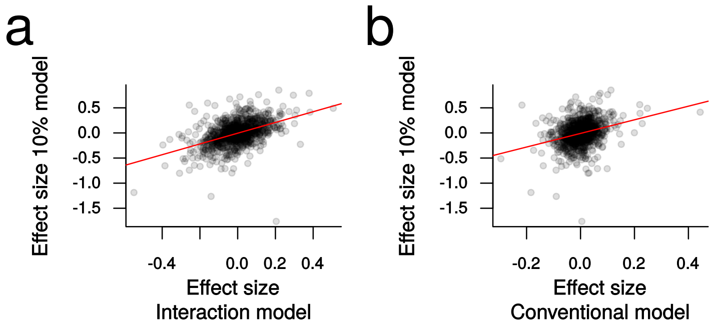

Figure S4: (a) The x-axis shows the eQTL effect sizes recovered by the interaction model, the y-axis shows the effect sizes calculated by a conventional model using only the 10% of breast cancer samples with the highest estimated cancer cell content. (b) The x-axis shows the effect sizes recovered by the conventional model (on the full dataset), rather than the interaction model. Globally, the eQTL effects estimated by the interaction model more closely match those for the model fit on the high-purity samples only ($r = 0.45$, $r_s = 0.43$ for interaction model and $r = 0.28$, $r_s = 0.3$ for conventional model ($P < 2.2 \times 10^{-16}$ in all cases)). For clarity, points are shown for 1,000 randomly chosen eQTLs.

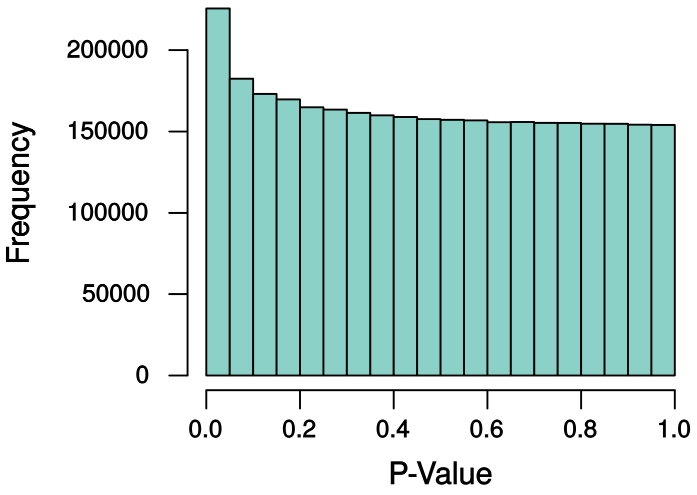

Figure S5: A histogram or the P-values for the difference in eQTL effect sizes between TCGA breast cancer and normal breast tissue from GTEx, calculated using EQ3 in Methods section of main text.

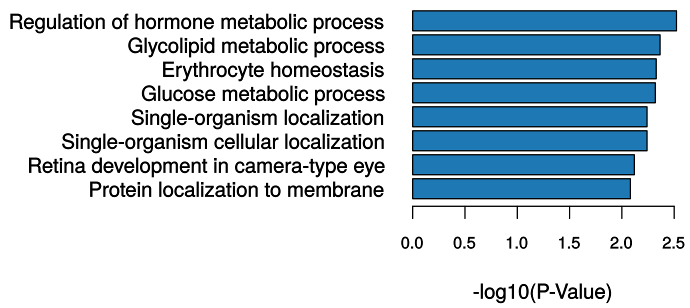

Figure S6: Bar graph of the lowest 8 P-values of 3,679 GO biological process tested; P-values are for the enrichment of genes whose eQTL profile changes between TCGA breast cancer (identified by the interaction model) and normal breast tissue in GTEx. This plot shows the enrichment for process when only eQTLs with a larger absolute value in breast cancer compared to normal breast tissue are considered.

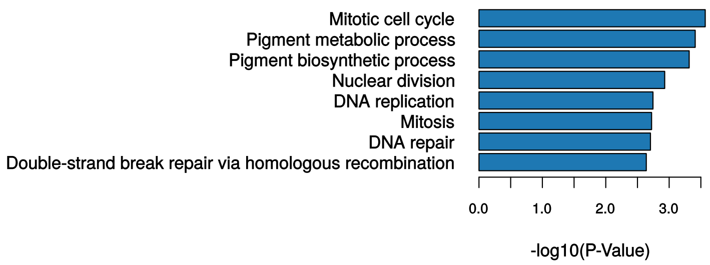

Figure S7: Bar graph of the lowest 8 P-values of 3,679 GO biological process tested; P-values are for the enrichment of genes whose eQTL profile changes between TCGA breast cancer (identified by the interaction model) and normal breast tissue in GTEx. This plot shows the enrichment for process when only eQTLs with a larger absolute value in normal (GTEx) breast tissue are considered.

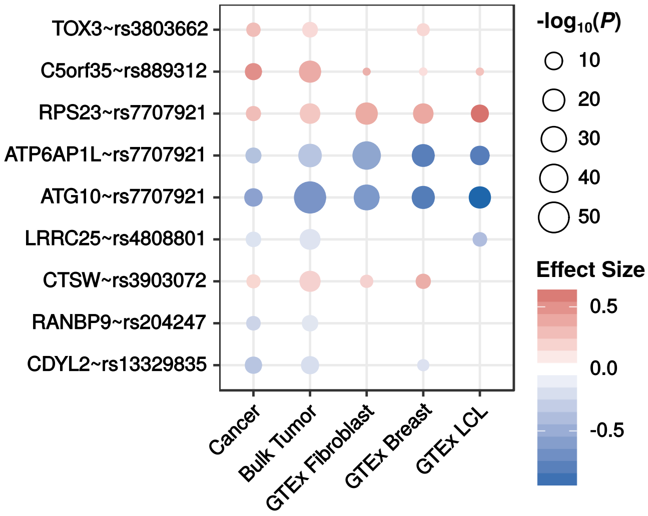

Figure S8: P-values and effect sizes for the nine eQTLs from GWAS significant SNPs that remain statistically significant ($FDR < 0.05$) when the interaction model is used (thus, these are high confidence cancer eQTLs). For each eQTL, the effect size is represented by the red-blue divergent color scale and the P-value by the size of the point; these are shown for cancer cells (i.e. the interaction model), TCGA bulk breast tumor (i.e. the conventional model), and fibroblast, breast and LCL tissues from GTEx. Associations with P $<$ 0.05 are shown.

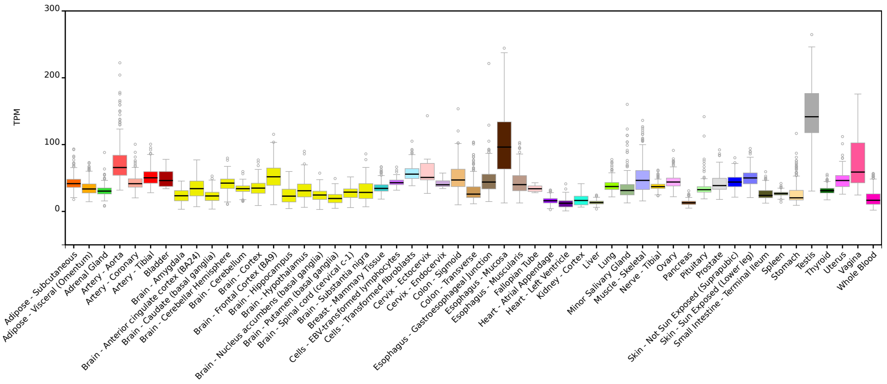

Figure S9: The expression levels of RANBP9 in humans. The y-axis shows the TPM expression estimates derived from various tissues of GTEx (x-axis). Figure generated using GTEx Analysis Release V7 (dbGaP Accession phs000424.v7.p2).

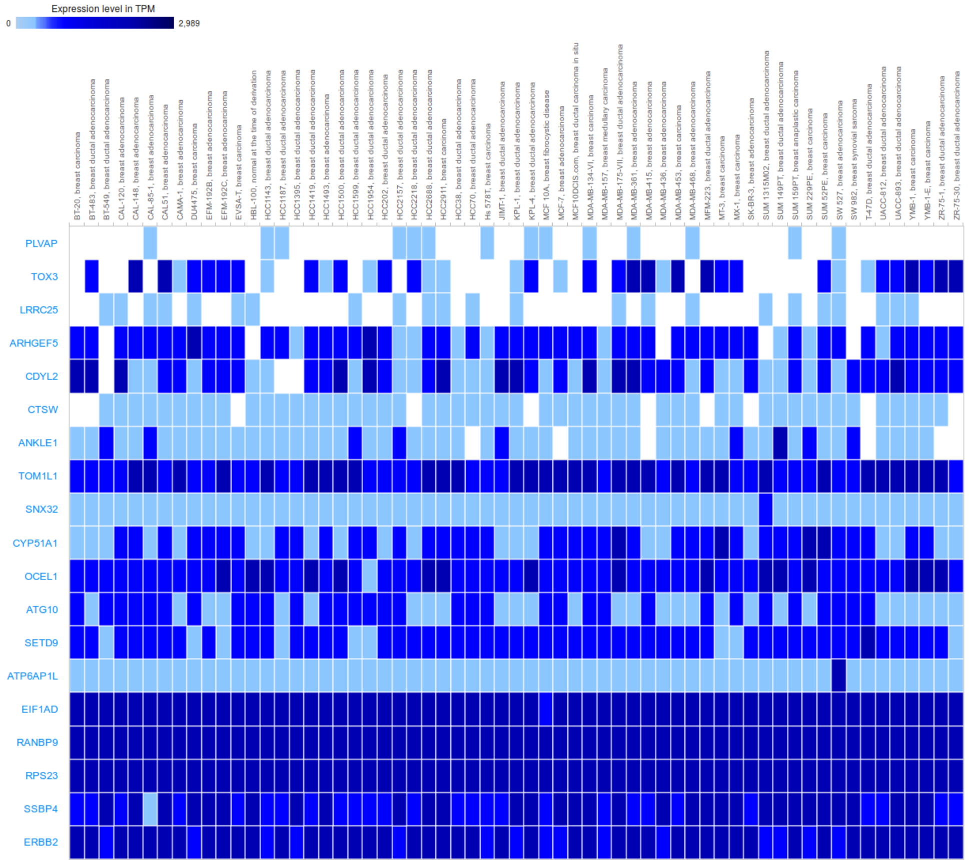

Figure S10: TPM expression estimates for select genes in breast cancer cell lines generated by RNA-seq. This data was generated as part of Klijn et al. (PMID:25485619). The genes shown are for the genes that are eQTL targets in breast cancer (i.e. the genes in Fig. 5(b)) and the genes that are eQTLs in bulk breast tumor, but are no longer even nominally significant in breast cancer (Supplementary Figure 8). The figure was generated using the EBI Expression Atlas.

Tissue	Samples	Beta	p-value	post-prob
Esophagus - Mucosa	358	-0.0927	2.0e-8	1.00
Artery - Aorta	267	-0.174	3.9e-5	0.989
Muscle - Skeletal	491	-0.0727	1.5e-3	0.962
Lung	383	-0.0864	0.01	0.764
Spleen	146	-0.141	0.02	0.725
Vagina	106	-0.0943	0.07	0.472
Whole Blood	369	-0.0475	0.03	0.418
Uterus	101	-0.0999	0.2	0.360
Artery - Tibial	388	-0.0492	0.1	0.259
Pancreas	220	-0.0595	0.2	0.245
Pituitary	157	-0.0614	0.3	0.209
Brain - Spinal cord (cervical c-1)	83	-0.0389	0.7	0.188
Brain - Hippocampus	111	-0.0280	0.6	0.176
Cells - EBV-transformed lymphocytes	117	-0.0331	0.7	0.173
Brain - Hypothalamus	108	-0.0325	0.6	0.141
Liver	153	-0.0114	0.9	0.133
Small Intestine - Terminal Ileum	122	0.0332	0.7	0.107
Stomach	237	-0.0231	0.5	0.0950
Brain - Amygdala	88	-0.00190	1	0.0930
Brain - Anterior cingulate cortex (BA24)	109	-0.0295	0.5	0.0860
Minor Salivary Gland	85	0.0220	0.8	0.0760
Prostate	132	-0.0200	0.8	0.0540
Adrenal Gland	175	0.0292	0.6	0.0470
Brain - Substantia nigra	80	0.0362	0.5	0.0370
Artery - Coronary	152	-0.00668	0.9	0.0360
Skin - Not Sun Exposed (Suprapubic)	335	-0.00432	0.9	0.0230
Brain - Frontal Cortex (BA9)	118	0.00591	0.9	0.0210
Breast - Mammary Tissue	251	0.00135	1	0.0190
Brain - Cerebellar Hemisphere	125	0.0664	0.3	0.0180
Brain - Putamen (basal ganglia)	111	0.143	0.06	0.0160
Brain - Cerebellum	154	0.0979	0.1	0.0130
Heart - Atrial Appendage	264	0.0328	0.4	0.0110
Cells - Transformed fibroblasts	300	0.0131	0.7	0.00700
Ovary	122	0.155	0.02	0.00600
Esophagus - Muscularis	335	0.0227	0.4	0.00500
Brain - Nucleus accumbens (basal ganglia)	130	0.244	1.7e-3	0.00400
Adipose - Visceral (Omentum)	313	0.0445	0.3	0.00300
Heart - Left Ventricle	272	-0.00538	0.9	0.00300
Esophagus - Gastroesophageal Junction	213	0.0228	0.5	0.00200
Brain - Caudate (basal ganglia)	144	0.100	0.07	0.00
Adipose - Subcutaneous	385	0.0933	4.2e-3	0.00
Brain - Cortex	136	0.0655	0.1	0.00
Testis	225	0.0629	0.01	0.00
Colon - Transverse	246	0.0581	0.08	0.00
Nerve - Tibial	361	0.0559	0.1	0.00
Colon - Sigmoid	203	0.0472	0.2	0.00
Skin - Sun Exposed (Lower leg)	414	0.0164	0.5	0.00
Thyroid	399	0.0138	0.7	0.00

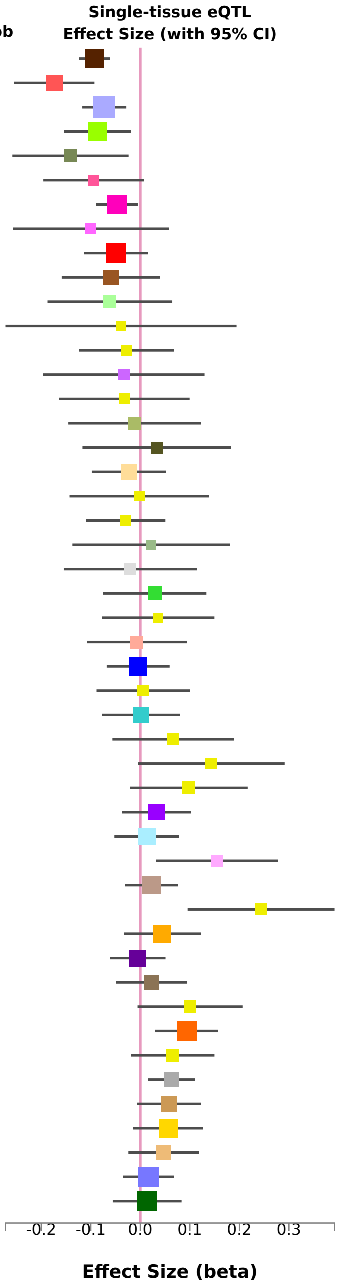

Figure S11: A table summarizing the P-values and effect sizes obtained for the RANBP9 eQTL rs204247 across all GTEx tissues. The "post-prob" column is the posterior probability of an eQTL existing in this tissue obtained from a cross-tissue Bayesian analysis (Han and Eskin, PLOS Genetics, referenced in main text). This figure was generated from GTEx Portal using GTEx Release V7.

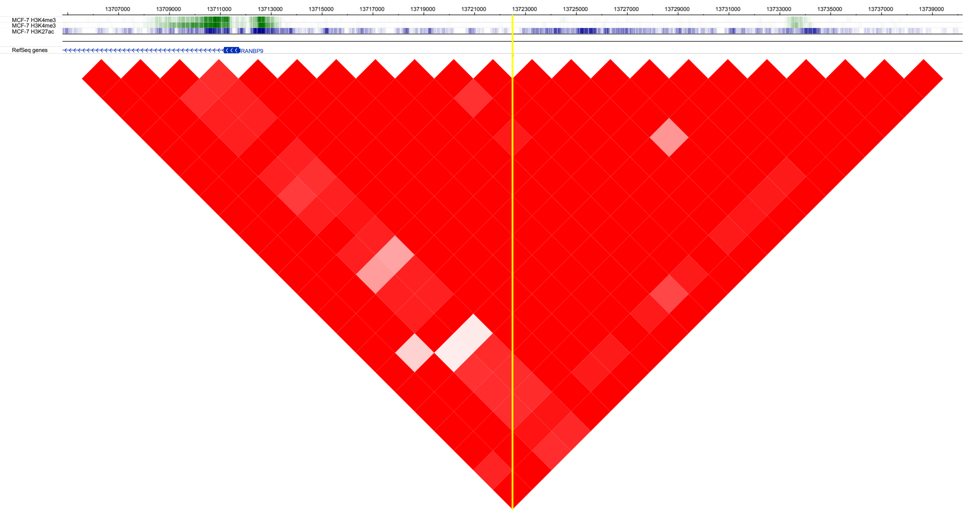

Figure S12: A genome browser plot showing the locus on human chromosome 6 that contains the RANBP9 transcription start site (HG19 co-ordinates). Shown in green are tracks for H3K4me3, a histone modification that is a canonical mark of active promoter. Shown in blue is H3K27ac, a histone acetylation modification that is a canonical mark of active enhancers. These tracks data were generated on an MCF7 breast cancer cell line as part of the ENCODE project. The linkage disequilibrium (LD) in the region is shown at the bottom of the plot. This was generated using release 27 of the Hapmap CEU data (Utah residents with northern and western European ancestry). Red squares indicate an R^2 value of 1, and white corresponds to an R^2 of 0. The vertical yellow line shows the location of the eQTL, rs204247. rs204247 is tagging the RANBP9 promoter and several putative enhancers. The initial version of this plot was created using the WashU Genome Browser.

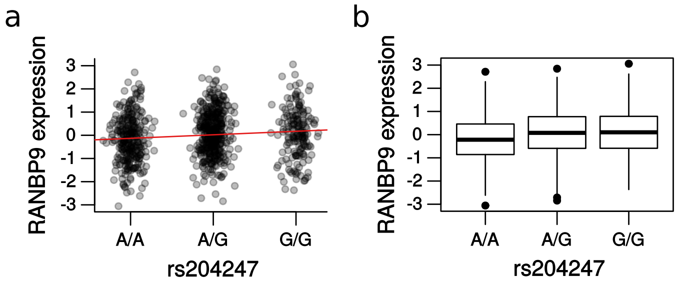

Figure S13: The association of rs204247 and RANBP9 in TCGA breast cancer. Note that the expression values on the y-axis are for bulk tumor gene expression. The P-value for this association from the interaction model was $P = 1.9 \times 10^{-4}$ $(FDR = 0.01)$, with the same direction-of-effect as shown. Note that G is the risk allele for breast cancer.

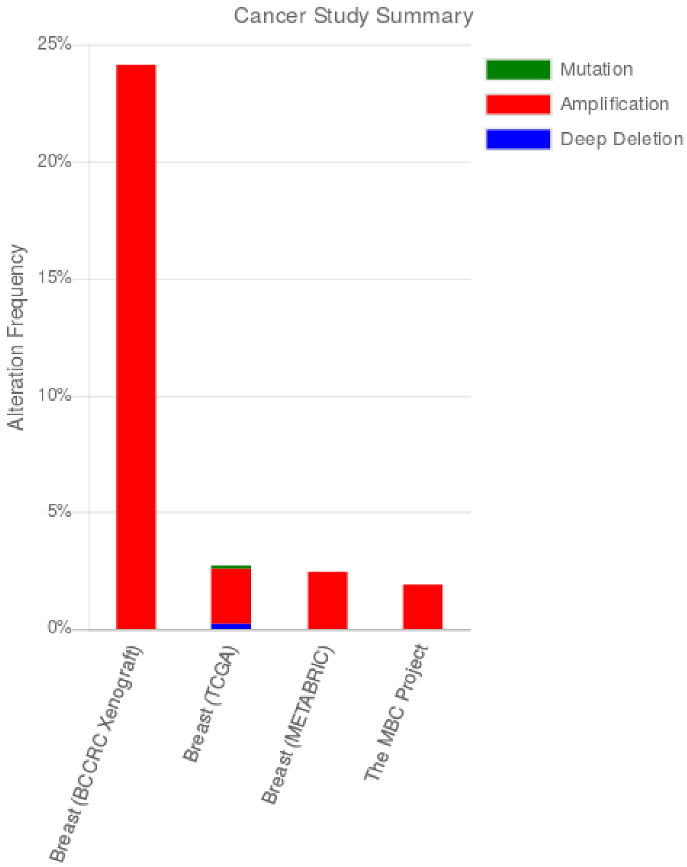

Figure S14: Plot generated using cBioPortal showing that RANBP9 is amplified in a subset of breast cancer patients in large breast cancer genomics studies.

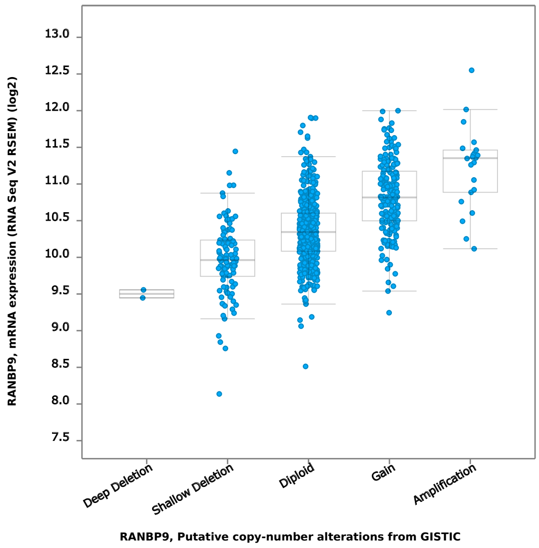

Figure S15: Plot generated using cBioPortal showing that amplification or gain of RANBP9 changes its expression in a large cohort of 1,080 TCGA breast cancer patients. Putative copy-number calls on 1,080 cases was determined using GISTIC 2.0.

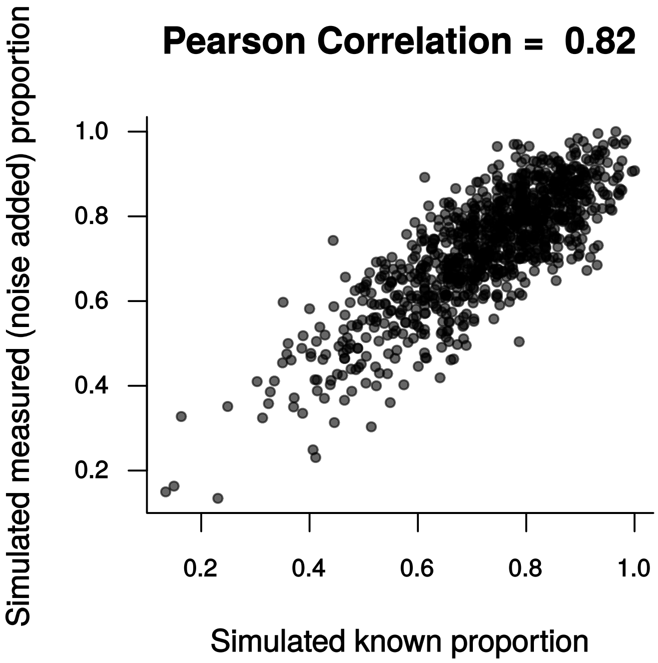

Figure S16: Correlation between the simulated true tumor purity estimates (x-axis), which were used to create the simulated mixture of cancer and normal expression data and the tumor purity estimates with noise added, which were used in the "interaction models" used to recover the cancer specific eQTL effect. These correlations are similar to the accuracy with which tumor purity could be expected to be estimated in real data.
