# Supplementary material for: Cancer expression quantitative trait loci (eQTLs) can be determined from heterogeneous tumor gene expression data by modeling variation in tumor purity
Source: Genome Biol. 2018 Sep 11;19:130. doi: 10.1186/s13059-018-1507-0 (PMC6131897; doi:10.1186/s13059-018-1507-0)
Supplement: Supplementary file 4 — Reviewer reports and Author’s response to reviewers. (DOCX 46 kb) [file 13059_2018_1507_MOESM4_ESM.docx]

**First round of review**

**Reviewer 1**

**Are the methods appropriate to the aims of the study, are they well described, and are necessary controls included?**
Mostly yes. But see the Reviewer's comments for details.

**Are the conclusions adequately supported by the data shown?**

Mostly yes. But see the Reviewer's comments for details.

**Are sufficient details provided to allow replication and comparison with related analyses that may have been performed?**

Yes. But the analysis pipeline should be included as supplementary information for reproducibility.

**Does the method perform better than existing methods (as demonstrated by direct comparison with available methods)?**

No: The authors should compare their method and results with other studies.

**Is the method likely to be of broad utility? Is any software component easy to install and use?**

Yes. The analysis pipeline should be included as supplementary information.

**Is the paper of broad interest to others in the field, or of outstanding interest to a broad audience of biologists?**

Yes: Overall, this study addressed an important issue regarding design and interpretations of results of cancer eQTL studies, which could be of broad interest.

**Comments to authors**

In the manuscript, 'Cancer cell specific eQTL effects can be identified from bulk tumor gene expression data by modeling tumor purity' Geeleher, Huang, and colleagues have developed a statistical framework to identify cell-type dependent eQTL effects from bulk tissue expression data and apply that framework to the TCGA data to identify tumor cell-specific eQTLs. Applying this framework they classify cancer-associated risk variants into six classes of eQTLs based on whether they are present in only tumor or benign tissues, or both, and conclude that about one-third of breast cancer risk variants detected based on uncorrected analysis of bulk tumor expression, could be confidently attributed to tumor cells. Overall, this study addressed an important issue regarding design and interpretations of results of cancer eQTL studies, which could be of broad interest. I have several specific comments.

1. The power to detect the six classes of eQTLs depends on tumor cell fraction, effect sizes of eQTLs, and sample size. Figure 1c and d show specific examples from simulated data, but a more systematic power analysis is warranted. The results for different classes of eQTLs based on TCGA data should be discussed accordingly.

2. The authors discussed the work of Li et al [3], but chose not to include somatic copy number/methylation in the model. Tumor genomes have frequent copy number alterations and CpG methylation changes, which affect gene expression patterns and contribute to gene expression variation within a cohort. It is unclear whether not including these important factors in the model limited their ability to detect eQTLs in tumor cells, driving the conclusion 'Only about one third of breast cancer risk variants, identified as eQTLs from an uncorrected analysis of bulk tumor expression, could be confidently attributed to cancer cells." Thus, it will be important to determine to what extent the key results are affected when allelic copy number/ploidy and promoter methylation adjusted expression estimates are incorporated in the model. 
The authors should also compare their method and results with other studies such as Li et al.

3. It will be important to validate key results in an independent cohort (e.g. METABRIC). 

4. Breast cancer has several different subtypes as identified by the TCGA, METABRIC and other studies. A subtype specific analysis is warranted to determine if some of the identified eQTLs are specific to certain breast cancer subtypes. 

5. Previous studies have linked genetic variants with clinical outcomes in breast cancer. Do the variants from the 6 different eQTL classes show differences in terms of association with clinical outcome in the TCGA? eQTLs not detected in cancer cells may still affect disease progression and clinical outcome. For instance, immune system plays a major role in prognosis. TCGA also has data on tumor-infiltrating lymphocyte cell fraction. It will be worth investigating to what extent the GTEx LCL eQTLs are also identified in the tumor-infiltrating lymphocyte cell fraction, and if such eQTLs have clinical relevance for cancer patients. 

Finally, the analysis pipeline should be included as supplementary information.

**Reviewer 2**

**Are the methods appropriate to the aims of the study, are they well described, and are necessary controls included?**

Yes

**Are the conclusions adequately supported by the data shown?**

Yes, but may be toned down somewhat

**Are sufficient details provided to allow replication and comparison with related analyses that may have been performed?**

Yes

**Is the method likely to be of broad utility? Is any software component easy to install and use?**

Yes, this methods holds the potential to be of brad utility to analyze eQTLs in tumor tissues of varying tumor percentages.

**Is the paper of broad interest to others in the field, or of outstanding interest to a broad audience of biologists?**

Yes: New method that appears to perform better than previous methods

**Comments to authors**

This manuscript describes a novel statistical method to define tumor specific eQTLs in heterogenous tumor samples, by using an interaction term for tumor purity * genotypes in the eQTL analysis and extrapolating the effect size to a sample with 100% tumor cells. The FDR dropped from 11.1% to 3.3% in their simulations indicating an improvement of the model. They then use the TCGA BRCA dataset (n=894 samples) to assess ~3.6M eQTLs (for genes +/- 500kb) and identify 57,189 significant cis-eQTL associations using a conventional eQTL analysis model. Using the interaction/extrapolation model, this number drops to 8,833 eQTLs that are attributed to cancer cells. This is an interesting paper that provides an important approach as to how to optimize eQTL analysis in tumor samples with ranging tumor percentages. However, there are important drawbacks that need improvement:

1. My main concern with the approach taken is manuscript is that the eQTL analysis does not appear to be adjusted for copy number variation in the TCGA BRCA samples. Although genotype data is based on blood derived DNA, the expression data from tumor samples will be influenced by deletions and amplifications in the breast tissue samples used. How can the authors distinguish between expression differences influenced by germline genotypes and those that occur because of copy number changes in the tumor samples? As an example of this potential problem, according to the authors, RANBP9 is amplified in ~13% of breast cancer. Is the eQTL due to copy number variation or is this a real eQTL? 

2. It would be very informative to perform regular eQTL analysis by restricting the TCGA BRCA samples to those with high tumor percentage to see if this recapitulates or supports the findings from the interaction analysis using all samples. From Figure 2a it appears that most samples have tumor purity over 70% so this restriction should not result in a large drop in statistical power.

3. Page 9, lines 9-11: "This eQTL likely arises from tumor-infiltrating stromal and immune cells, …" and Page 11, lines 33-36 "However, 9 of these eQTLs were not even nominally significant (P > 0.05) when extrapolated to cancer cells using the interaction model suggesting these are strong candidates for eQTLs arising from infiltrating normal cells." 

It may be too simplistic to assign eQTLs that are not observed in the interaction model to infiltrating normal cells. Similar strong language is used throughout the manuscript that assumes a little too strongly that the interaction model is "the gold standard". With a sensitivity of 58% in the simulation, that is not the case. Other reasons for these negative findings could be that i) copy number alterations in the tumor expression data do not appear to be considered in the simulations and analysis, and ii) even with 894 samples, there are limitations in statistical power. 

4. Page 11, line 31: I have a similar concern here as for item above: "However, 9 of these eQTLs were not even nominally significant (P > 0.05) when extrapolated to cancer cells using the interaction model, suggesting these are strong candidates for eQTLs arising from infiltrating normal cells. Indeed, all of these 9 associations were significant in at least one of fibroblast, breast or LCL tissues in GTEx, in all cases with the same directionality as the eQTL effect estimated from bulk tumor expression using the conventional model. Using the interaction model, another 9 of these 24 SNPs could be confidently assigned to cancer cells (FDR < 0.05). Five of these were strong cross-tissue eQTLs in GTEx …"

In the absence of having a microdissected breast tumor eQTL dataset, the authors may be making too many assumptions about cancer specific eQTLs vs. those in normal cells. Also, discussing normal breast tissue as "infiltrating normal cells" is misleading. The strong cross-tissue eQTLs (page 11 lines 45-52) are assigned to the breast cancer cells but the 9 not seen in the interaction model (page 11, lines 31-41) were assigned to normal infiltrating cells as they are also seen in fibroblasts, normal breast or LCL's. This seems contradictory.

As to negative finding, these eQTLs may have been important at earlier stages in development in the normal epithelial tissue - or are important only in the presence of a specific stimulus that is not captured in the dataset being used. It would be good to discuss better other potential causes for seeing or not detecting tumor eQTLs. 

5. There is too much discussion in results section. This should be minimized and/or moved to the discussion section.

**Reviewer 3**

**Are the methods appropriate to the aims of the study, are they well described, and are necessary controls included?**

See comments below.

**Are the conclusions adequately supported by the data shown?**

See comments below.

**Are sufficient details provided to allow replication and comparison with related analyses that may have been performed?**

See comments below.

**Is the method likely to be of broad utility? Is any software component easy to install and use?**

See comments below.

**Is the paper of broad interest to others in the field, or of outstanding interest to a broad audience of biologists?**

Yes: see comments below

**Comments to author:**

The manuscript by Geeleher P et al. presents very interesting data showing that most reported eQTLs discovered using tumour expression cannot be attributed to cancer cells but rather to tumour-infiltrating normal cells. These findings are extremely relevant and highlight a shortcoming of current eQTL mapping strategies in cancer. The authors have used breast cancer as a case-study but these findings could be applied to other cancer types and other complexe disease, where most tissues used for eQTL mapping are a mixture of cells.
The manuscript is clearly written and easy to follow. The results are clearly presented.
Although this study is of great interest to the field, I do not believe I have the expertise to evaluate the mathematical and statistical approaches that were developed. For this reason, I do not believe I can comment on the acceptability of this manuscript.

**Author’s Response to Reviewers**

REVIEWER COMMENTS 

Reviewer #1 

Reviewer #1, comment 1: 
Reviewer #1: In the manuscript, 'Cancer cell specific eQTL effects can be identified from bulk tumor gene expression data by modeling tumor purity' Geeleher, Huang, and colleagues have developed a statistical framework to identify cell-type dependent eQTL effects from bulk tissue expression data and apply that framework to the TCGA data to identify tumor cell-specific eQTLs. Applying this framework they classify cancer-associated risk variants into six classes of eQTLs based on whether they are present in only tumor or benign tissues, or both, and conclude that about one-third of breast cancer risk variants detected based on uncorrected analysis of bulk tumor expression, could be confidently attributed to tumor cells. Overall, this study addressed an important issue regarding design and interpretations of results of cancer eQTL studies, which could be of broad interest. I have several specific comments. 
1. The power to detect the six classes of eQTLs depends on tumor cell fraction, effect sizes of eQTLs, and sample size. Figure 1c and d show specific examples from simulated data, but a more systematic power analysis is warranted. The results for different classes of eQTLs based on TCGA data should be discussed accordingly. 


Reviewer #1, response 1: 
We have now included discussion on statistical power, and how this differs between the “conventional” and “interaction” model for various classes of eQTLs. We have included a new figure (Supplementary Figure 1) and appended information on power calculations to Supplementary Tables 1 and 2 and commented on this in the main text (pg. 6 line 122-124). Notably, power is intrinsically dependent on some unknown factors, in particular, the level of shared eQTL signal in cancer and tumor-associated normal cells. This means the most important metric to compare different approaches remains the false discovery rate, which as we have demonstrated is not adequately controlled by a conventional model. 


Reviewer #1, comment #2: 
2. The authors discussed the work of Li et al [3], but chose not to include somatic copy number/methylation in the model. Tumor genomes have frequent copy number alterations and CpG methylation changes, which affect gene expression patterns and contribute to gene expression variation within a cohort. It is unclear whether not including these important factors in the model limited their ability to detect eQTLs in tumor cells, driving the conclusion 'Only about one third of breast cancer risk variants, identified as eQTLs from an uncorrected analysis of bulk tumor expression, could be confidently attributed to cancer cells." Thus, it will be important to determine to what extent the key results are affected when allelic copy number/ploidy and promoter methylation adjusted expression estimates are incorporated in the model. 
The authors should also compare their method and results with other studies such as Li et al. 

Reviewer#1, response #2: 
We have now compared our results to those achieved when controlling for copy number and methylation status, as per Li et al. This step does not substantially change our original findings and does not change our conclusions. We have also added more discussion on the merits and limitations of the approach presented by Li et al. Notably, subsequent to Li et al., published in 2013, effective methods for controlling for expression heterogeneity in eQTL analysis have become standard (i.e. PEER, which was used in our analysis) because they already capture most of the variability introduced by hidden confounders like copy number and methylation. However, PEER also allows computational efficiency to be dramatically improved, because only one inversion of the design matrix (by far the most computationally expensive step in eQTL model fitting) is required per SNP and its entire set of possible target genes, rather than a matrix inversion being required for every SNP-gene pair, as is the case if different covariates (copy number status, promoter methylation) are included for each gene. In addition, controlling for methylation and copy number will cause a loss in power under a situation where there is a shared mechanism for a methylation and expression QTL (e.g. PMID:29476079) or where there is a tendency for some copy number variants to develop on a specific germline background (e.g. PMID:28188128). Given these issues, our main text primarily focuses on the original results obtained after analysis using PEER; however, we have also included the results from these additional analyses which include a covariate for copy number and methylation (main text pg. 8, line 180-182, Supplementary Tables 4 & 5; Supplementary Figure 3) 


Reviewer # 1, comment #4: 
3. It will be important to validate key results in an independent cohort (e.g. METABRIC). 

Reviewer #1, response #4: 
Per reviewer’s suggestion, we have applied the models to the METABRIC data, an independent breast cancer patient cohort, where the findings were consistent with those observed in TCGA (see main text (Results) pg. 11, lines 236-251, (Methods) pg. 21 lines 480-500) 


Reviewer #1, comment #5: 
4. Breast cancer has several different subtypes as identified by the TCGA, METABRIC and other studies. A subtype specific analysis is warranted to determine if some of the identified eQTLs are specific to certain breast cancer subtypes. 

Reviewer #1, response #5: 
We have reported results for samples broken down by subtypes (HER2+, Luminal A, Luminal B and basal-like). eQTL profiles are very similar between subtypes. Statistically significant association identified in the subtype-specific analyses are also almost always significant in the analysis of all breast cancer samples pooled. These results have been included in Supplementary Tables 4 & 5. 


Reviewer #1, comment #6 
5. Previous studies have linked genetic variants with clinical outcomes in breast cancer. Do the variants from the 6 different eQTL classes show differences in terms of association with clinical outcome in the TCGA? eQTLs not detected in cancer cells may still affect disease progression and clinical outcome. For instance, immune system plays a major role in prognosis. TCGA also has data on tumor-infiltrating lymphocyte cell fraction. It will be worth investigating to what extent the GTEx LCL eQTLs are also identified in the tumor-infiltrating lymphocyte cell fraction, and if such eQTLs have clinical relevance for cancer patients. 

Reviewer #1, response #6 
Although the questions raised by the reviewer are indeed interesting, it is somewhat different to the main point of our manuscript, which was to show tumor-associated normal cells need to be considered when conducting cancer eQTL analysis. Nonetheless, as the reviewer points out, previous studies have searched for common genetic variants prognostic of breast cancer outcome. For example: 

(a) “Germline variation in ADAMTSL1 is associated with prognosis following breast cancer treatment in young women” Kadalayil et al., Nature Communications (2017).
(b) “CHST9 rs1436904 genetic variant contributes to prognosis of triple-negative breast cancer”, Yuan et al., Scientific Reports (2017). 
(c) “Identification of inherited genetic variations influencing prognosis in early-onset breast cancer”, Rafiq et al., Cancer Research (2013). 

However, these studies did not achieve genome-wide significance (P < 5 × 10-8), nor to our knowledge have these results been replicated. Thus, previously reported associations between germline variation and survival in breast cancer are tenuous. Consistent with this, we did not find genome-wide significant associations with survival in the TCGA cohort. Generally speaking, we feel rigorously addressing this question would likely involve a separate study, as it involves very complex analyses, with numerous possible confounders and is somewhat beyond the scope of our current objective. 


Reviewer #1, comment #7: 
Finally, the analysis pipeline should be included as supplementary information. 

Reviewer #1, response #7: 
We have posted the analysis pipeline on GitHub: https://github.com/paulgeeleher/cancerEqtls and Open Science Framework: https://osf.io/z7uyp/ 


Reviewer #2 

Reviewer #2: This manuscript describes a novel statistical method to define tumor specific eQTLs in heterogeneous tumor samples, by using an interaction term for tumor purity * genotypes in the eQTL analysis and extrapolating the effect size to a sample with 100% tumor cells. The FDR dropped from 11.1% to 3.3% in their simulations indicating an improvement of the model. They then use the TCGA BRCA dataset (n=894 samples) to assess ~3.6M eQTLs (for genes +/- 500kb) and identify 57,189 significant cis-eQTL associations using a conventional eQTL analysis model. Using the interaction/extrapolation model, this number drops to 8,833 eQTLs that are attributed to cancer cells. This is an interesting paper that provides an important approach as to how to optimize eQTL analysis in tumor samples with ranging tumor percentages. However, there are important drawbacks that need improvement: 


Reviewer #2, comment #1: 
1. My main concern with the approach taken is manuscript is that the eQTL analysis does not appear to be adjusted for copy number variation in the TCGA BRCA samples. Although genotype data is based on blood derived DNA, the expression data from tumor samples will be influenced by deletions and amplifications in the breast tissue samples used. How can the authors distinguish between expression differences influenced by germline genotypes and those that occur because of copy number changes in the tumor samples? As an example of this potential problem, according to the authors, RANBP9 is amplified in ~13% of breast cancer. Is the eQTL due to copy number variation or is this a real eQTL? 

Reviewer #2, response #1: 
As stated above, we have now included an analysis that (similar to Li et al.) includes covariates for copy number change and for methylation. Because we had already controlled for expression heterogeneity using PEER, these covariate were largely redundant, make little difference to the final results and do not affect our conclusions (see main text pg. 8, line 180-182 Supplementary Table 4 & 5; Supplementary Figure 3). 


Reviewer #2, comment #2: 
2. It would be very informative to perform regular eQTL analysis by restricting the TCGA BRCA samples to those with high tumor percentage to see if this recapitulates or supports the findings from the interaction analysis using all samples. From Figure 2a it appears that most samples have tumor purity over 70% so this restriction should not result in a large drop in statistical power. 

Reviewer #2, response #3: 
We thank the reviewer for sharing this interesting idea. We tested this in simulated data and in TCGA breast cancer samples. In simulated data, restricting to very pure samples does indeed improve the true false discovery rate (FDR), which is well controlled when restricting to the purest samples (i.e. the top 10%). However, this is at the cost of greatly reduced sensitivity to detect true cancer eQTL associations, compared to the interaction model. In TCGA breast cancer samples, restricting to the 10% most “pure” samples recovers only 248 significant associations (FDR < 0.05). Interestingly however, when we globally compared all eQTL effects recovered by the “top 10% model” to both the interaction model and the conventional model, the results of the interaction model are far more similar to those obtained by the “top 10% model” (r = 0.447, 95% CI = 0.446-0.448 for the interaction model; r = 0.299, 95% CI = 0.299-0.3 for the conventional model). This provides additional support that the interaction model is more accurately estimating eQTL effects in cancer cells and these findings are now presented in Results (main text pg. 7 lines 151-153, pg. 10 lines 212-217, Supplementary Figures 2 & 4). 


Reviewer #2, comment #4: 
3. Page 9, lines 9-11: "This eQTL likely arises from tumor-infiltrating stromal and immune cells, …" and Page 11, lines 33-36 "However, 9 of these eQTLs were not even nominally significant (P > 0.05) when extrapolated to cancer cells using the interaction model suggesting these are strong candidates for eQTLs arising from infiltrating normal cells." 

It may be too simplistic to assign eQTLs that are not observed in the interaction model to infiltrating normal cells. Similar strong language is used throughout the manuscript that assumes a little too strongly that the interaction model is "the gold standard". With a sensitivity of 58% in the simulation, that is not the case. Other reasons for these negative findings could be that i) copy number alterations in the tumor expression data do not appear to be considered in the simulations and analysis, and ii) even with 894 samples, there are limitations in statistical power. 


Reviewer #2, response #4: 
As stated above, we do not simply assume that not observed eQTLs in tumors are necessarily driven by infiltrating normal cells, nor was it ever our intention that the results were interpreted this way. Therefore, we have edited the wording to be more explicit as stated in the response above. See this text from the Discussion: “Additionally, our model, or any such model, cannot prove a non-association. It is incorrect to conclude that tumor eQTLs that cannot be attributed to cancer cells are definitely not eQTLs in cancer cells, or are certainly eQTLs in tumor-associated normal cells. The correct interpretation is that there is no statistical evidence for this eQTL in cancer cells at the current sample size and given factors such as the accuracy with which the data were measured. Notably, cancer eQTLs identified by the interaction model may still be eQTLs in other tumor-associated normal cell types; these should not be interpreted as exclusively-cancer eQTLs.” Also as stated above, our findings do not change after re-analysis with CNV as a covariate. The reviewer is, of course, correct that even with 894 samples there are still limitations in statistical power, particularly when the additional burden of identifying eQTLs in different cell types is considered. 


Reviewer #2, comment #5: 
4. Page 11, line 31: I have a similar concern here as for item above: "However, 9 of these eQTLs were not even nominally significant (P > 0.05) when extrapolated to cancer cells using the interaction model, suggesting these are strong candidates for eQTLs arising from infiltrating normal cells. Indeed, all of these 9 associations were significant in at least one of fibroblast, breast or LCL tissues in GTEx, in all cases with the same directionality as the eQTL effect estimated from bulk tumor expression using the conventional model. Using the interaction model, another 9 of these 24 SNPs could be confidently assigned to cancer cells (FDR < 0.05). Five of these were strong cross-tissue eQTLs in GTEx …" 

In the absence of having a microdissected breast tumor eQTL dataset, the authors may be making too many assumptions about cancer specific eQTLs vs. those in normal cells. Also, discussing normal breast tissue as "infiltrating normal cells" is misleading. The strong cross-tissue eQTLs (page 11 lines 45-52) are assigned to the breast cancer cells but the 9 not seen in the interaction model (page 11, lines 31-41) were assigned to normal infiltrating cells as they are also seen in fibroblasts, normal breast or LCL's. This seems contradictory. As to negative finding, these eQTLs may have been important at earlier stages in development in the normal epithelial tissue - or are important only in the presence of a specific stimulus that is not captured in the dataset being used. It would be good to discuss better other potential causes for seeing or not detecting tumor eQTLs. 

Reviewer #2, response #5: 
The reviewer makes some excellent points. To address these, firstly, throughout we have replaced the term “tumor-infiltrating normal cells” with “tumor-associated normal cells”, as we agree with the reviewer that “tumor-infiltrating” is not always accurate in the context it had been used. Also, at the end of the introduction, we clarified exactly what the terms like “tumor-associated normal cells” are meant to convey in the context of this manuscript (as this type of terminology is not always used consistently in the literature). Again, it was never our intention to assume that eQTLs no longer significant using the “interaction model” are necessarily assigned to normal cells. Though GTEx is suggestive of this for a great deal of these eQTLs, this assignment cannot be made using the current data and methodologies (see above). 


Reviewer #2, comment #6: 
5. There is too much discussion in results section. This should be minimized and/or moved to the discussion section. 

Reviewer #2, response #6: 
We have moved more text from Results to Discussion. 


Reviewer #3: 
The manuscript by Geeleher P et al. presents very interesting data showing that most reported eQTLs discovered using tumour expression cannot be attributed to cancer cells but rather to tumour-infiltrating normal cells. These findings are extremely relevant and highlight a shortcoming of current eQTL mapping strategies in cancer. The authors have used breast cancer as a case-study but these findings could be applied to other cancer types and other complexe disease, where most tissues used for eQTL mapping are a mixture of cells. 
The manuscript is clearly written and easy to follow. The results are clearly presented. 
Although this study is of great interest to the field, I do not believe I have the expertise to evaluate the mathematical and statistical approaches that were developed. For this reason, I do not believe I can comment on the acceptability of this manuscript. 

Reviewer #3, response 
We thank the reviewer for their positive comments. 
